# Supplementary material for: Salicylic Acid Protects Sweet Potato Seedlings from Drought Stress by Mediating Abscisic Acid-Related Gene Expression and Enhancing the Antioxidant Defense System
Source: Int J Mol Sci. 2022 Nov 26;23(23):14819. doi: 10.3390/ijms232314819 (PMC9736078; doi:10.3390/ijms232314819)
Supplement: Supplementary file 1 [file ijms-23-14819-s001.zip › ijms-2023802-supplementary.pdf]

Supplemental Tables

Table S1. Effects of drought stress on growth traits of sweet potato

	Vine length(cm)		Dry matter weight(g)		Leaf area(cm^2)	
	ZS77	ZS13	ZS77	ZS13	ZS77	ZS13
CK	25.69±2.18	27.42±2.21	27.74±3.36	29.67±3.28	33.95±4.10	36.73±4.28
C0	14.81±1.67	15.46±1.42	15.35±1.78	16.43±1.03	19.58±1.35	20.71±1.22

The dry matter weight and leaf area were given for per plant.

Table S2. The primers used for real-time RT-PCR.

NCED3-like	
Forward primer	ATCTGCGCTTCACACTCCTC
Reverse primer	ACTTTCGGGTGGGCAATCAT
β-actin	
Forward primer	ACTCAGTGGCGGGACTAC
Reverse primer	CTGTGAACAATTGACGGACC
